# Confined van der Waals Epitaxial Growth of Two-Dimensional Large Single-Crystal In_2_Se_3_ for Flexible Broadband Photodetectors

**DOI:** 10.34133/2019/2763704

**Published:** 2019-03-19

**Authors:** Lei Tang, Changjiu Teng, Yuting Luo, Usman Khan, Haiyang Pan, Zhengyang Cai, Yue Zhao, Bilu Liu, Hui-Ming Cheng

**Affiliations:** ^1^Shenzhen Geim Graphene Center, Tsinghua-Berkeley Shenzhen Institute, Tsinghua University, Shenzhen 518055, China; ^2^Shenzhen Institute for Quantum Science and Engineering and Department of Physics, Southern University of Science and Technology, Shenzhen 518055, China; ^3^Shenzhen Key Laboratory of Quantum Science and Engineering, Shenzhen 518055, China; ^4^Shenyang National Laboratory for Materials Sciences, Institute of Metal Research, Chinese Academy of Sciences, Shenyang 110016, China

## Abstract

The controllable growth of two-dimensional (2D) semiconductors with large domain sizes and high quality is much needed in order to reduce the detrimental effect of grain boundaries on device performance but has proven to be challenging. Here, we analyze the precursor concentration on the substrate surface which significantly influences nucleation density in a vapor deposition growth process and design a confined micro-reactor to grow 2D In_2_Se_3_ with large domain sizes and high quality. The uniqueness of this confined micro-reactor is that its size is ~10^2^-10^3^ times smaller than that of a conventional reactor. Such a remarkably small reactor causes a very low precursor concentration on the substrate surface, which reduces nucleation density and leads to the growth of 2D In_2_Se_3_ grains with sizes larger than 200 *μ*m. Our experimental results show large domain sizes of the 2D In_2_Se_3_ with high crystallinity. The flexible broadband photodetectors based on the as-grown In_2_Se_3_ show rise and decay times of 140 ms and 25 ms, efficient response (5.6 A/W), excellent detectivity (7×10^10^ Jones), high external quantum efficiency (251%), good flexibility, and high stability. This study, in principle, provides an effective strategy for the controllable growth of high quality 2D materials with few grain boundaries.

## 1. Introduction

**Figure 1:**
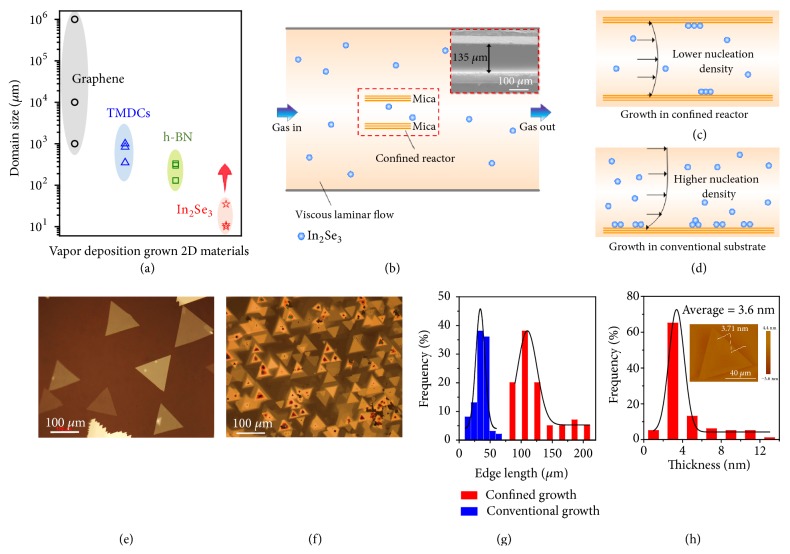
Growth of 2D In_2_Se_3_ with large domain sizes in a confined micro-reactor. (a) A summary of the domain sizes of 2D materials including graphene, TMDCs, h-BN, and In_2_Se_3_ grown by conventional vapor phase deposition. (b) Schematic of the confined micro-reactor for the growth of 2D In_2_Se_3_ on mica with large domain sizes. The inset shows a side-view SEM image of the reactor, which is composed of two stacked mica sheets with an average distance of 135 *μ*m between them. (c, d) Schematics showing different gas flow behaviors in the (c) confined and (d) conventional growth methods. (e, f) Optical images of In_2_Se_3_ crystals grown on the mica substrates with confined and conventional growth. (g, h) Statistical data of the edge length and thickness of In_2_Se_3_ grown using the two methods. The inset in (h) shows a typical AFM image of 2D In_2_Se_3_ grown in the confined micro-reactor.

Two-dimensional (2D) materials have been considered promising candidates for miniaturized and high-performance electronic and optoelectronic devices due to their atomically flat and ultrathin nature and their lack of dangling bonds. Grain boundaries and defects in 2D materials can reduce charge transport, [1] mechanical, [2–4] and thermal properties [5]. Therefore, the controllable growth of 2D materials with large domain sizes and high quality with few grain boundaries and defects is important in order to achieve good device performance. To this end, different vapor deposition methods have been developed to grow 2D materials with large domain sizes, such as epitaxial growth on special substrates (e.g., sapphire [6] or mica [7, 8]), reducing nucleation density by locally feeding the precursors [9, 10], and passivating active sites during growth [11]. As summarized in Figure 1(a), the current domain sizes of semi-metallic graphene range from micrometers to meters [9, 12, 13], while semiconducting transition metal dichalcogenides (TMDCs) [14–16] and insulating hexagonal boron nitride (h-BN) have sizes up to hundreds of micrometers or larger [17–19]. Recently, another group of 2D materials with the structure A_2_B_3_, where A is a group III element and B is a group VI element, such as indium selenide (In_2_Se_3_, its structure is shown in [Supplementary-material supplementary-material-1]), has attracted increasing interest. First, In_2_Se_3_ has a direct bandgap of 1.36 eV [20], which is close to that of silicon (1.10 eV). Second, although some monolayer TMDCs like MoTe_2_ also have a direct bandgap of 1.10 eV, they become indirect bandgap materials as the number of layers increases [21], while In_2_Se_3_ is a direct bandgap material regardless of its thickness [22, 23]. Third, unlike black phosphorus which is also a direct bandgap 2D material, thin In_2_Se_3_ flakes are very stable in air, which is very important for practical applications. Recently, there has been pioneering work on the use of 2D In_2_Se_3_ in piezoelectronics [24, 25], optoelectronics [26], and photovoltaics [27]. We note that these reported 2D In_2_Se_3_ materials have small domain sizes (from a few to tens of micrometers, Figure 1(a) and [Supplementary-material supplementary-material-1]) [28–30]. Therefore, controllable growth of high quality 2D In_2_Se_3_ with large domains is of great importance to further advance the use of 2D In_2_Se_3_.

To grow 2D materials with large domains, the control of nucleation density is critical, as has been extensively studied in graphene growth [31]. Usually, in the vapor deposition process, the gas flow is controlled to be viscous laminar in order to obtain a constant atmosphere [32]. As shown in [Supplementary-material supplementary-material-1], there is a velocity gradient of gas in a reactor, and the velocity gradually decreases to zero near the substrate surface, forming a stagnant layer above the substrate. Due to the fact that the precursor concentration on the substrate surface can influence the nucleation density, controlling the precursor concentration on the surface is important to control the nucleation of the 2D materials ([Supplementary-material supplementary-material-1]). By analyzing the vapor phase deposition process, we have obtained the following formula for the precursor concentration on the surface (*C*_s_):

(1) $$\begin{array}{c} C_{s}\sim \frac{1}{1+k_{s}/\sqrt{d}}C_{g} \end{array}$$

where *C*_g_ is the precursor concentration in the gas phase, *k*_s_ is the surface reaction rate, and* d* is the characteristic size of the growth reactor ([Supplementary-material supplementary-material-1]). Based on formula (1), *C*_s_ decreases as* d* decreases, causing a lower concentration of precursor on the surface and consequently there is a lower nucleation density of 2D materials in a smaller growth reactor.

Based on the above analyses and previous works [33, 34], we designed a confined micro-reactor to greatly reduce the size of the growth space so as to grow 2D In_2_Se_3_ with large domain sizes. Specifically, the micro-reactor is composed of two slices of mica with a face-to-face stacking feature. First, the size of growth space is effectively reduced by two to three orders, from 10^5^-10^4^ *μ*m to 10^2^ *μ*m, so that the nucleation density of 2D In_2_Se_3_ is reduced. Second, the mica, used as a substrate for van der Waals epitaxial growth, benefits the growth of 2D In_2_Se_3_ with large domain sizes and thin thickness, because the atomically smooth surface and lack of dangling bonds greatly reduce strain from lattice mismatch between the mica and In_2_Se_3_. As a result, in the confined micro-reactor, high-quality In_2_Se_3_ with domain sizes larger than 200 *μ*m has been grown on the mica. Moreover, direct growth on flexible mica facilitates the fabrication of flexible photodetectors which have a response time of 140 ms for rise and 25 ms for decay, high responsivity (5.6 A/W), high detectivity (7×10^10^ Jones), and large external quantum efficiency (EQE, 251%). After bending 1000 times, the photodetector showed a steady photocurrent with an 80% retention, indicating good flexibility.

## 2. Results 

As shown in Figure 1(b), the confined micro-reactor is composed of two slices of freshly cleaved mica, stacked face to face. The space (*d*) between them is only 140 *μ*m, as seen from a side-view scanning electron microscope (SEM) image (inset of Figure 1(b)). This is two to three orders of magnitude smaller than the diameter of the quartz tube in conventional growth experiments (~20-50 mm). To grow the In_2_Se_3_, In_2_Se_3_ powder was used as the precursor, and the two slices of mica served as a micro-reactor (see details in Supplementary Materials and [Supplementary-material supplementary-material-1]). Because of the design of this reactor (Figure 1(c)), we obtained 2D In_2_Se_3_ with large domain sizes. As shown in Figure 1(e), the products are triangular with sharp edges and large domain sizes. In contrast, in In_2_Se_3_ grown in a conventional reactor (*d* > 20 mm, Figure 1(d)), the surface of mica is dirtier and the In_2_Se_3_ crystals grown have smaller domain sizes as shown in Figure 1(f). Moreover, the edge length and thickness of In_2_Se_3_ samples grown in two different reactors were measured by atomic force microscopy (AFM, Figures 1(g), 1(h), and [Supplementary-material supplementary-material-1]). The results show that the average edge length and thickness of In_2_Se_3_ are 110 *μ*m and 3.6 nm, respectively, grown in the space-confined micro-reactor. This average edge length of 110 *μ*m is much larger than that of In_2_Se_3_ grown in the conventional substrate, with an average edge length of 40 *μ*m (Figure 1(g)).

**Figure 2:**
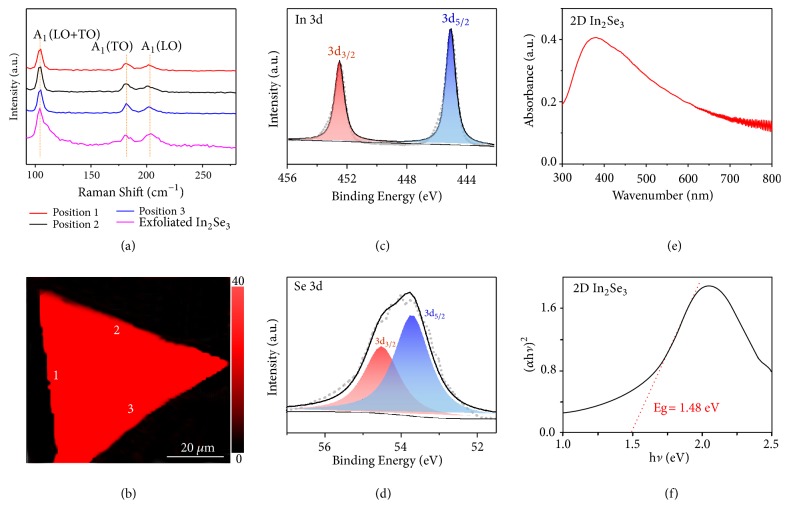
Spectroscopic characterization of the as-grown 2D In_2_Se_3_ in the confined micro-reactor. (a) Raman spectra of In_2_Se_3_. The three typical Raman peaks at ~108, ~180, and ~203 cm^−1^ are attributed to the A_1_(LO+TO), A_1_(TO), and A_1_(LO) phonon modes of In_2_Se_3_. (b) Raman intensity map of a 2D In_2_Se_3_ flake with a thickness of ~10 nm, showing good uniformity_._ (c, d) XPS spectra of the In 3d and Se 3d in In_2_Se_3_. (e) UV-Vis-NIR absorption spectrum of the In_2_Se_3_ grown on mica. (f) h*ν* versus (*α*h*ν*)^2^ plot of the sample.

Insight into the structure and chemical composition of the In_2_Se_3_ was obtained from spectroscopic characterization.  Figure 2(a) shows Raman spectra of the In_2_Se_3_ collected at different positions using a 532 nm excitation laser. All spectra show three typical peaks at ~108, 180, and 203 cm^−1^, corresponding to the A_1_(LO+TO), A_1_(TO), and A_1_(LO) phonon modes of In_2_Se_3_, respectively. We compared the Raman spectra with those of 2D exfoliated In_2_Se_3_ by using a scotch tape (pink curve, Figure 2(a)) and data from recent literature [25], and they all match well. To confirm the uniformity of the In_2_Se_3_, we also performed Raman mapping (Figure 2(b)), which indicated good uniformity and homogeneity of the material grown in the confined micro-reactor. The X-ray photoelectron spectroscopy (XPS) spectrum of In 3d shows two peaks located at 452.20 and 444.80 eV from In 3d_3/2_ and In 3d_5/2_, originating from In_2_Se_3_ (Figure 2(c)). Similarly, the XPS spectrum of Se 3d shows two peaks at 54.80 and 53.80 eV from Se 3d_3/2_ and Se 3d_5/2_, also attributed to In_2_Se_3_ (Figure 2(d)) [35]. In addition, the XPS results show that the In_2_Se_3_ has good stoichiometry with an In/Se atomic ratio close to 2:3 (Figure S5). Ultraviolet-visible-near infrared absorption spectroscopy (UV-Vis-NIR) was used to study the optical bandgap of the In_2_Se_3_ (Figure 2(e)) which is obtained from the following equation:

(2) $$\begin{array}{c} {\left(\;\alpha E\;\right)}^{1/n}=C\left(\;E-E_{g}\;\right) \end{array}$$

where *α* is the effective absorption coefficient of the material, *E* is incident photon energy, *C* is a constant, *E*_g_ is the optical bandgap of the material, and n denotes the nature of the sample transition, which equals 2 for indirect bandgap materials and 0.5 for direct bandgap materials. *E*_g_ was calculated to be 1.48 eV for n = 0.5 (Figure 2(f)), which is close to the value reported in previous work (1.36 eV) [20]. In total, all these spectroscopy results indicate that the synthesized materials are uniform In_2_Se_3_ with good stoichiometry and optical quality.

**Figure 3:**
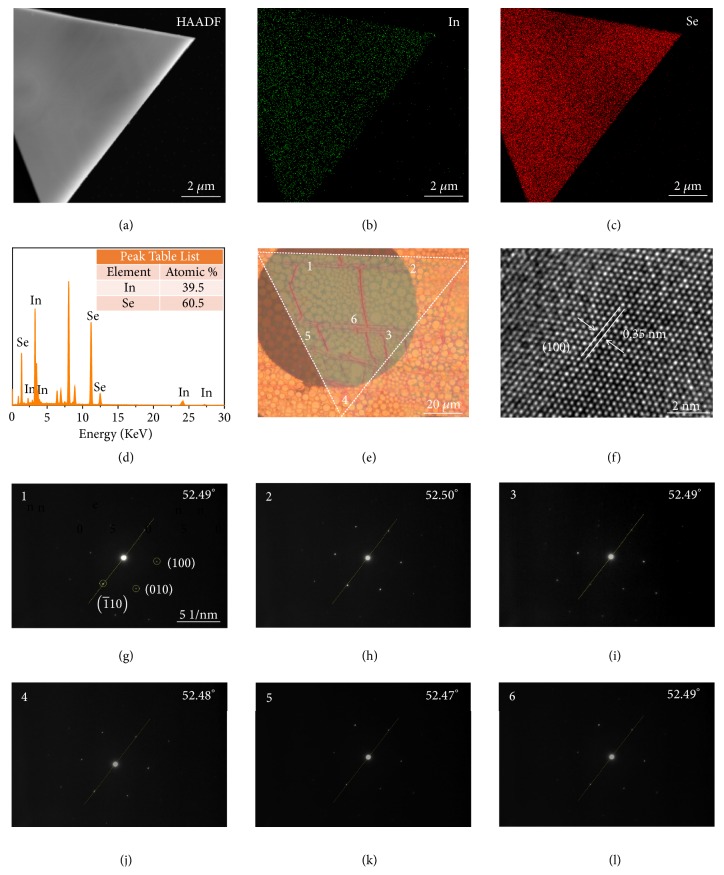
Microstructural characterization of the In_2_Se_3_. (a) HAADF image of In_2_Se_3_ flake transferred onto a TEM grid. (b, c) EDS maps of In and Se for this In_2_Se_3_ flake. (d) An EDS spectrum of the sample, which shows an atomic ratio of 2:3 (In:Se). (e, f) Optical microscope (e) and HRTEM (f) images of a In_2_Se_3_ flake transferred onto a TEM grid. (g–l) SEAD patterns obtained from the six positions labeled 1–6 on the transferred sample in (e).

We also investigated the structure and crystal quality of the material using X-ray diffraction (XRD) and transmission electron microscopy (TEM). The XRD pattern of the In_2_Se_3_ grown on the mica substrate in the confined reactor was compared with the patterns of bulk In_2_Se_3_ and mica, and it was found that, except for the peaks from the mica substrate, all peaks originated from In_2_Se_3_ ([Supplementary-material supplementary-material-1]). Additionally, the In_2_Se_3_ has a high crystallinity because the peaks are very sharp. The TEM samples were prepared using a modified transfer method assisted by polydimethylsiloxane (PDMS) and polymethyl methacrylate (PMMA, see details in the Supporting Information). After repeated trial and error, we developed this method to transfer 2D In_2_Se_3_ onto arbitrary substrates such as plastics, SiO_2_/Si, a copper grid, and indium tin oxide (ITO) glass (Figures [Supplementary-material supplementary-material-1]-[Supplementary-material supplementary-material-1]). Raman spectra show that the transferred samples have very similar spectra to the as-grown ones (Figures [Supplementary-material supplementary-material-1]-[Supplementary-material supplementary-material-1]), indicating that negligible damage was done to the samples during transfer.  Figure 3(a) shows a high-angle annular dark-field scanning TEM (HAADF-STEM) image of a triangular 2D In_2_Se_3_ flake. The corresponding energy dispersive X-ray spectroscopy (EDS) elemental maps show the uniform distributions of In and Se atoms in the In_2_Se_3_ (Figures 3(b) and 3(c)). In addition, quantitative analysis of the EDS results (Figure 3(d)) shows an In:Se atomic ratio of 2:3, in good agreement with the XPS results.  Figure 3(e) is an optical image of a In_2_Se_3_ flake transferred onto a TEM copper grid. The high-resolution TEM (HRTEM) image confirms that it has high quality, and the lattice spacing of 0.35 nm corresponds to the In_2_Se_3_ (100) lattice planes (Figure 3(f)) [40]. Selected area electron diffraction (SAED, Figures 3(g)–3(l)) patterns were recorded from six positions in this flake (marked 1–6 in Figure 3(e)) far away from each other. It can be seen that all the patterns have hexagonal symmetry with the same crystallographic orientation, confirming that it is a single crystal domain. All these results confirm that the 2D In_2_Se_3_ grown in the confined micro-reactor is high quality and highly crystalline.

**Figure 4:**
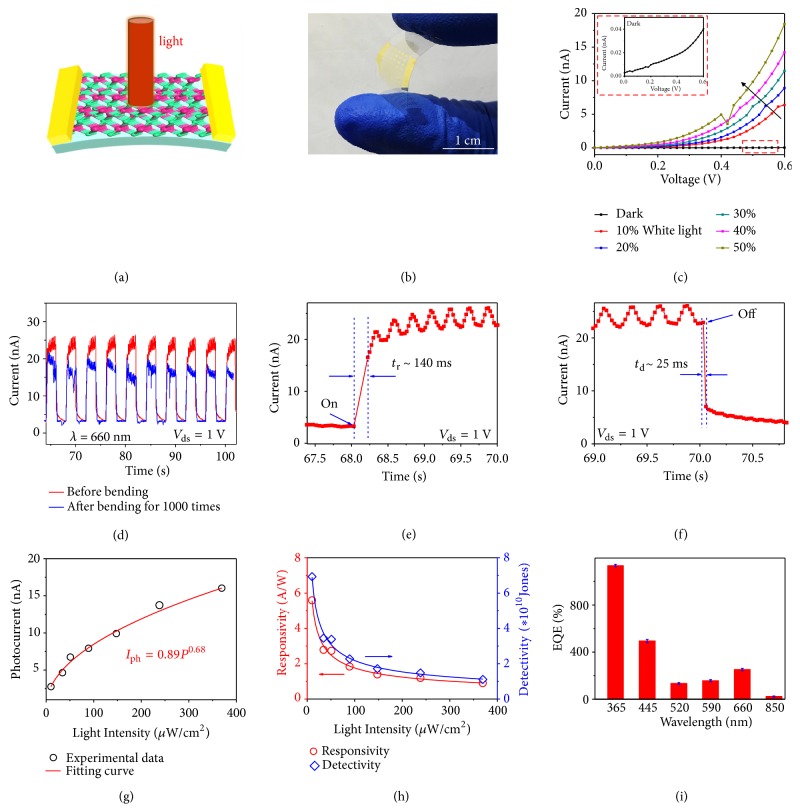
2D In_2_Se_3_ based flexible photodetectors. (a) Schematic of a flexible photodetector on a mica substrate using a 2D In_2_Se_3_ flake with a thickness of ~7 nm as the channel material. (b) Photo showing the bendable device of the 2D In_2_Se_3_ on a mica substrate. (c) Current under white light with different power intensities. (d) Time-resolved photoresponse of the same device with 660 nm light before and after bending 1000 times. (e, f) The exponential curves of the rise and decay times. (g) Photocurrent versus power intensity for 660 nm incident light. (h) Responsivity and detectivity of the device under 660 nm incident light with different powder intensities. (i) The relationship between EQE and different incident light wavelengths (UV-Vis-NIR range). All data were measured at 300 K, under atmospheric conditions and *V*_ds_ = 1 V.

Generally, compared to monolayer semiconductors, few-layer ones with direct and appropriate bandgaps are promising candidates for photodetectors due to their enhanced light absorption, lower energy loss during photo-electron conversion, and better carrier transfer between source and drain [41]. TMDCs like MoS_2_ cannot satisfy all the requirements at the same time because it has an indirect-to-direct bandgap transition when the number of layers decreases from a few layers to a monolayer, so that few-layer MoS_2_ has a low photo-electron conversion efficiency and monolayer MoS_2_ has weak light absorption.[39, 42] Hence, for MoS_2_ and many other TMDCs, a sacrifice of properties is unavoidable to get a balance between light absorption and photo-electron conversion efficiency [43]. Fortunately, In_2_Se_3_ is a direct bandgap material regardless of its thickness and shows clear advantages in optoelectronics [22, 23]. Therefore, we fabricated two terminal devices on a flexible mica substrate using 2D In_2_Se_3_ with large domain sizes and high quality as the channel material and studied their photoresponse behavior (Figures 4(a) and 4(b)). Several key parameters including dark current (*I*_dark_), rise time (*t*_r_), decay time (*t*_d_), responsivity (*R*), detectivity (*D*^*∗*^), and EQE were systematically investigated under 660 nm incident light.  Figure 4(c) shows that the photocurrent (*I*_ph_ defined as *I*_ph_ = *I*_light_ − *I*_dark_) increases with the incident light power, and the largest on/off ratio reaches 460. Unlike other narrow bandgap semiconductors, In_2_Se_3_ shows a large photo-induced on-off ratio, due to its appropriate bandgap and high quality, i.e., lack of defects. In order to investigate the response speed and stability of the photodetector, time-resolved photoresponse measurements were performed by turning on/off the incident light with a chopper, while a high-speed oscilloscope was used to monitor the device current. As shown in Figure 4(d), the In_2_Se_3_ photodetector remains stable under several on/off light switching events (660 nm incident light at *V*_ds_ = 1 V) with a nearly constant “on-state” current of ~25 nA (Figure 4(d)), with *t*_r_ and *t*_d_ calculated to 140 ms and 25 ms, respectively (Figures 4(e) and 4(f)). We note that the response time is much slower than commercial Si based photodetectors (~5.9 *μ*s). Further engineering and optimization of the quality of materials and interface cleanness of devices should improve the response time. Note that, after bending 1000 times, the current remains steady with a retention of >80% (with an “on-state” current of ~ 20 nA, Figure 4(d)). We also shined 850 and 940 nm incident light on the device and it showed good stability (Figures [Supplementary-material supplementary-material-1] and [Supplementary-material supplementary-material-1]). It is well known that most semiconductors are sensitive to visible light, but few show an appropriate responsivity and response speed to NIR light. These results suggest that the In_2_Se_3_ is a promising candidate for high-performance photodetectors in the UV-Vis-NIR region. Meanwhile, when we changed the power of the incident 660 nm light (*P*_in_), different *I*_ph_ values were obtained ([Supplementary-material supplementary-material-1]). The relationship between *I*_ph_ and *P*_in_ is fitted by *I*_ph_ = a*P*^*α*^. In our experiments, the parameters a and *α* were calculated to be 0.89 and 0.68, respectively (Figure 4(g)). Moreover, as shown in Figure 4(h), we obtained a remarkable responsivity of 5.6 A/W (under* P* = 10.2 *μ*W cm^−2^, *V*_ds_ = 1 V), which was calculated from the following formula:

(3) $$\begin{array}{c} R=\frac{I_{ph}}{P_{in}S} \end{array}$$

where *R* is the responsivity and *S* is the effective area of the photodetector. This responsivity is 10^3^ times higher than the reported value of multilayer MoS_2_-based phototransistors (around 7.5 × 10^−3^ A/W), presumably because few-layer MoS_2_ has an indirect bandgap [37]. In addition, based on the following formula,

(4) $$\begin{array}{c} D^{*}=\frac{RS^{1/2}}{{\left({2eI}_{dark}\right)}^{1/2}} \end{array}$$

where *D*^*∗*^ is detectivity and e is the charge of an electron, we calculated the detectivity and found an identical trend with responsivity. Impressively, its maximum *D*^*∗*^ reaches 7 × 10^10^ Jones (under *P* = 10.2 *μ*W cm^−2^, *V*_ds_ = 1 V) as shown in Figure 4(h), and this value is three orders of magnitude higher than that of MoS_2_ photodetectors [37]. This detectivity is superior to the vast majority of reported 2D material-based phototransistors. Finally, we calculated EQE based on the different light photoresponses ([Supplementary-material supplementary-material-1]), using the following:

(5) $$\begin{array}{c} EQE=\frac{Rhc}{\left(e\lambda \right)} \end{array}$$

where *h* is Planck's constant,* c* the velocity of light, and *λ* the wavelength of the incident light. The EQE of the In_2_Se_3_ photodetector (*V*_ds_ = 1 V) was calculated to be 1135% at 365 nm, 495% at 445 nm, 127% at 520 nm, 154% at 590 nm, 251% at 660 nm, and 18% at 850 nm. These results suggest that few-layer In_2_Se_3_ grown on mica, as a direct bandgap semiconductor, is suitable for use in flexible broadband photodetectors.

A comparison of the performance of photodetectors using our 2D In_2_Se_3_ and other 2D materials is shown in Table 1. Overall, the combination of high responsivity, high detectivity, and flexibility makes In_2_Se_3_ a promising material for flexible broadband photodetectors. We believe that the large size, high quality, and thin thickness of the In_2_Se_3_ can prolong the photo-excited carrier lifetime and result in a high photoresponsivity. In addition, the use of a mica substrate which has an atomically flat surface may reduce trap states at the interface between the In_2_Se_3_ and the mica substrate, leading to long photo-excited carrier lifetime. As shown from the formula R ∝ *I*_ph_ ∝ Δ*σ* = q(*μ*_n_ + *μ*_p_)(Δp)_0_exp⁡(−t/*τ*), it is clear that a longer photo-excited carrier lifetime (*τ*) leads to improved photoconductivity (*σ*), a larger photocurrent, and better responsivity.

## 3. Discussion

In summary, we designed a confined micro-reactor that greatly reduces the size of the growth space and thus the nucleation density of 2D materials. As a result, we achieved the growth of 2D In_2_Se_3_ with large domain sizes and high quality. Because of the large domain size, high quality, and layer-independent direct bandgap, we have been able to fabricate In_2_Se_3_-based flexible photodetectors, which have a fast response speed, and high responsivity, detectivity, and EQE. The strategy used here could potentially shed light on the growth of other 2D materials, facilitating their application in a wide-range of devices.

## 4. Materials and Methods

*Materials and Chemicals*. In_2_Se_3_ powder (99.99%, Alfa Aesar, USA), fluorphlogopite mica ([KMg_3_(AlSi_3_O_10_)F_2_], Tiancheng Fluorphlogopite Mica Co., Ltd., China), polydimethylsiloxane (PDMS) tape (200 *μ*m thickness, Hangzhou Bao Er De New Materials Technology Co., Ltd., China), polymethyl methacrylate (PMMA, 950 K, ALLRESIST, AR-P 672.045, Germany), acetone, and ethanol (AR, Shanghai Macklin Biochemical Co., Ltd., China) were used as received.

*Vapor Phase Growth of 2D In*_*2*_ *Se*_*3*_. In our experiments, growth was conducted in a homemade atmospheric pressure vapor deposition furnace equipped with a 1-inch diameter quartz tube (TF55035C-1, Lindberg/Blue M). The quartz boat containing In_2_Se_3_ powder was put at the center of the furnace, and two slices of freshly cleaved mica were placed downstream (8-10 cm), stacked face-to-face, and served as a confined micro-reactor for 2D In_2_Se_3_ growth. The furnace was heated to the growth temperature of 850°C, which was determined by the thermo-gravimetric analysis ([Supplementary-material supplementary-material-1]), with a ramp rate of 30°C min^−1^, and kept there for 5-30 min for the growth. Ar was introduced during the ramping and growth periods at a flow rate of 50-100 standard cubic centimeters per minute (sccm, with a purity of 99.99%). After growth, the furnace was cooled to room temperature under 50 sccm Ar. In the controlled growth experiments, 2D In_2_Se_3_ was grown on a freshly cleaved mica substrate placed in the same location but without the other mica sheet.

*Transfer of As-Grown 2D In*_*2*_ *Se*_*3*_ *onto a TEM Grid.* The TEM samples were prepared by transferring 2D In_2_Se_3_ using a PMMA and PDMS assisted transfer method [44]. First, PMMA solution was spin-coated onto the mica substrate with the grown In_2_Se_3_ (3000 rpm for 1 min). Second, the substrate was heated in air at 170°C for 5 min to form a PMMA film which served as a supporting layer and protected the In_2_Se_3_ in the following steps. Third, the PDMS tape was placed on the PMMA and the substrate was heated at 180°C for 5 min to make a strong bond between the PDMS and PMMA. Fourth, the PDMS/PMMA/In_2_Se_3_ was peeled off the mica substrate. Due to the hydrophobicity of PMMA and hydrophilicity of the mica, some water was introduced at the PMMA/mica interface to assist the separation of PMMA/In_2_Se_3_ and the mica substrate during the peeling-off. Fifth, the PDMS/PMMA/In_2_Se_3_ was attached to target substrates, followed by removing the PDMS tape. Finally, the PMMA was removed by hot acetone and the transferred In_2_Se_3_ sample was dried naturally in an ambient environment for further characterization.

*Characterization of 2D In*_*2*_ *Se*_*3*_. The side view of the space between the two mica sheets was checked by SEM (Hitachi SU8010, Japan). Optical images of the In_2_Se_3_ crystal were taken using an optical microscope (Carl Zeiss Microscopy, Germany). The thickness of the In_2_Se_3_ was determined by AFM (tapping mode, Bruker Dimension Icon, Germany). Raman spectroscopy was performed under a 532 nm laser excitation (Horiba LabRAB HR800, Japan). The laser spot was 1 *μ*m and the laser power on the sample surface was less than ~100 *μ*W. Structural and chemical analyses of the samples were performed by XRD (Cu K*α* radiation, *λ* = 0.15418 nm, Bruker D8 Advance, Germany), XPS (Thermo Scientific K-Alpha XPS, using Al (K*α*) radiation as a probe, USA), and TEM (FEI Tacnai F30, 300 kV acceleration voltage, USA) with an attached EDS unit. UV-Vis-NIR absorption was conducted to study the continuous In_2_Se_3_ films (Perkin-Elmer Lambda 950 spectrophotometer, USA).

*Device Fabrication and Measurements.* The as-grown In_2_Se_3_ sample was aligned with a shadow mask and titanium/gold (Ti/Au, 5 nm/50 nm) electrodes were then made by electron beam evaporation. Electrical measurements were conducted under a microprobe station and semiconductor property analyzer (Keithley 4200 SCS, USA) under ambient environment at room temperature. LEDs with different wavelengths ranging from 365 nm to 940 nm were used as incident light during photodetection measurements (CEL-LED535, LED Multiband and High-power Supply, China).

## Figures and Tables

**Table 1:** A comparison of the photoresponse of In_2_Se_3_ with other 2D materials.

Materials	Responsivity	Rise time	Decay time	Spectral	Type	Ref.
	(A/W)	(ms)	(ms)	range		
Graphene (on SiO_2_/Si)	1 × 10^−3^	10^−6^	NA	Visible	Rigid	[36]
MoS_2_ (on SiO_2_/Si)	7.5× 10^−3^	50	50	Visible	Rigid	[37]
BP (on SiO_2_/Si)	4.8 × 10^−3^	1	NA	Visible-NIR	Rigid	[38]
In_2_Se_3_ (on SiO_2_/Si)	2.5	NA	NA	Visible	Rigid	[28]
WSe_2_ (on PI)	0.92	900	2000	UV-NIR	Flexible	[39]
In_2_Se_3_ (on mica)	5.6	140	25	UV-NIR	Flexible	This work

## Supplementary Materials

Supplementary Materials
* Figure S1*. Crystal structure of layered In_2_Se_3_ with each layer composed of Se-In-Se-In-Se atomic sheets.* Figure S2*. Schematics showing viscous laminar flow in the vapor deposition process and related physical parameters.* Figure S3*. Thermo-gravimetric analysis of the In_2_Se_3_ source in an Ar atmosphere, and the temperature window for the growth of 2D In_2_Se_3_ in this work is shown by the green region.* Figure S4*. AFM images of the as-grown 2D In_2_Se_3_ on mica by confined growth.* Figure S5*. Survey XPS spectrum of as-grown 2D In_2_Se_3_ on mica.* Figure S6*. XRD patterns of 2D In_2_Se_3_ grown on mica (red) with reference patterns from a blank mica substrate (blue), bulk In_2_Se_3_ (green), and a simulated diffractogram (black).* Figure S7*. PDMS assisted transfer of 2D In_2_Se_3_ from a mica substrate onto different substrates.* Figure S8*. Time-resolved photoresponse of the 2D In_2_Se_3_ photodetector under 850 nm and 940 nm light.* Figure S9*. I–V curves of the 2D In_2_Se_3_ photodetector under 660 nm incident light with different power values.* Figure S10*. I–V curves of the 2D In_2_Se_3_ photodetector under different incident light wavelengths.

## References

[1] Cai Z., Liu B., Zou X., Cheng H.-M. (2018). Chemical vapor deposition growth and applications of two-dimensional materials and their heterostructures. *Chemical Reviews*.

[2] Huang M., Biswal M., Park H. J. (2018). Highly oriented monolayer graphene grown on a Cu/Ni(111) alloy foil. *ACS Nano*.

[3] Liu Y., Yakobson B. I. (2010). Cones, pringles, and grain boundary landscapes in graphene topology. *Nano Letters*.

[4] Cai X., Luo Y., Liu B., Cheng H.-M. (2018). Preparation of 2D material dispersions and their applications. *Chemical Society Review*.

[5] Peng L., Xu Z., Liu Z., Guo Y., Li P., Gao C. (2017). Ultrahigh thermal conductive yet superflexible graphene films. *Advanced Materials*.

[6] Zhang X., Choudhury T. H., Chubarov M. (2018). Diffusion-controlled epitaxy of large area coalesced WSe_2_ monolayers on sapphire. *Nano Letters*.

[7] Wu J., Yuan H., Meng M. (2017). High electron mobility and quantum oscillations in non-encapsulated ultrathin semiconducting Bi_2_O_2_Se. *Nature Nanotechnology*.

[8] Li J., Wang Z. X., Wen Y. (2018). High-performance near-infrared photodetector based on ultrathin Bi_2_O_2_Se nanosheets. *Advanced Functional Materials*.

[9] Wu T., Zhang X., Yuan Q. (2016). Fast growth of inch-sized single-crystalline graphene from a controlled single nucleus on Cu-Ni alloys. *Nature Materials*.

[10] Vlassiouk I. V., Stehle Y., Pudasaini P. R. (2018). Evolutionary selection growth of two-dimensional materials on polycrystalline substrates. *Nature Materials*.

[11] Wang M., Wu J., Lin L. (2016). Chemically engineered substrates for patternable growth of two-dimensional chalcogenide crystals. *ACS Nano*.

[12] Gao L., Ren W., Xu H. (2012). Repeated growth and bubbling transfer of graphene with millimetre-size single-crystal grains using platinum. *Nature Communications*.

[13] Xu X., Zhang Z., Dong J. (2017). Ultrafast epitaxial growth of metre-sized single-crystal graphene on industrial Cu foil. *Chinese Science Bulletin*.

[14] Chen W., Zhao J., Zhang J. (2015). Oxygen-assisted chemical vapor deposition growth of large single-crystal and high-quality monolayer MoS_2_. *Journal of the American Chemical Society*.

[15] Gao Y., Hong Y. L., Yin L. C. (2017). Ultrafast growth of high-quality monolayer WSe_2_ on Au. *Advanced Materials*.

[16] Yang T., Zheng B., Wang Z. (2017). Van der Waals epitaxial growth and optoelectronics of large-scale WSe_2_/SnS_2_ vertical bilayer p-n junctions. *Nature Communications*.

[17] Ji Y., Calderon B., Han Y. (2017). Chemical vapor deposition growth of large single-crystal Mono-, Bi-, Tri-layer hexagonal boron nitride and their interlayer stacking. *ACS Nano*.

[18] Wang L., Wu B., Liu H. (2017). Water-assisted growth of large-sized single crystal hexagonal boron nitride grains. *Materials Chemistry Frontiers*.

[19] Lu G., Wu T., Yuan Q. (2015). Synthesis of large single-crystal hexagonal boron nitride grains on Cu-Ni alloy. *Nature Communications*.

[20] Feng W., Zheng W., Gao F. (2016). Sensitive electronic-skin strain sensor array based on the patterned two-dimensional *α*-In_2_Se_3_. *Chemistry of Materials*.

[21] Sirota B., Glavin N., Krylyuk S., Davydov A. V., Voevodin A. A. (2018). Hexagonal MoTe_2_ with amorphous BN passivation layer for improved oxidation resistance and endurance of 2D field effect transistors. *Scientific Reports*.

[22] Ding W., Zhu J., Wang Z. (2017). Prediction of intrinsic two-dimensional ferroelectrics in In_2_Se_3_ and other III2-VI3 van der Waals materials. *Nature Communications*.

[23] Zheng Z. Q., Yao J. D., Yang G. W. (2016). Growth of centimeter-scale high-quality In_2_Se_3_ films for transparent, flexible and high performance photodetectors. *Journal of Materials Chemistry C*.

[24] Xue F., Zhang J., Hu W. (2018). Multidirection piezoelectricity in mono- and multilayered hexagonal *α*-In_2_Se_3_. *ACS Nano*.

[25] Zhou Y., Wu D., Zhu Y. (2017). Out-of-plane piezoelectricity and ferroelectricity in layered *α*-In_2_Se_3_ nanoflakes. *Nano Letters*.

[26] Zhai T., Fang X., Liao M. (2010). Fabrication of high-quality In_2_Se_3_ nanowire arrays toward high-performance visible-light photodetectors. *ACS Nano*.

[27] Bouchama I., Boudour S., Bouarissa N., Rouabah Z. (2017). Quantum and conversion efficiencies optimization of superstrate CIGS thin-films solar cells using In_2_Se_3_ buffer layer. *Optical Materials*.

[28] Lin M., Wu D., Zhou Y. (2013). Controlled growth of atomically thin In_2_Se_3_ flakes by van der Waals epitaxy. *Journal of the American Chemical Society*.

[29] Zhou J., Zeng Q., Lv D. (2015). Controlled synthesis of high-quality monolayered *α*-In_2_Se_3_ via physical vapor deposition. *Nano Letters*.

[30] Zheng W., Xie T., Zhou Y. (2015). Patterning two-dimensional chalcogenide crystals of Bi_2_Se_3_ and In_2_Se_3_ and efficient photodetectors. *Nature Communications*.

[31] Wang H., Xu X., Li J. (2016). Surface monocrystallization of copper foil for fast growth of large single-crystal graphene under free molecular flow. *Advanced Materials*.

[32] Bhaviripudi S., Jia X., Dresselhaus M. S., Kong J. (2010). Role of kinetic factors in chemical vapor deposition synthesis of uniform large area graphene using copper catalyst. *Nano Letters*.

[33] Wang X., Gong Y., Shi G. (2014). Chemical vapor deposition growth of crystalline monolayer MoSe_2_. *ACS Nano*.

[34] Cong C., Shang J., Wu X. (2014). Synthesis and optical properties of large-area single-crystalline 2D semiconductor WS_2_ monolayer from chemical vapor deposition. *Advanced Optical Materials*.

[35] Nelson A. J., Swartzlander A. B., Tuttle J. R., Noufi R., Patel R., Höchst H. (1993). Photoemission investigation of the electronic structure at polycrystalline CuInSe_2_ thin-film interfaces. *Journal of Applied Physics*.

[36] Xia F., Mueller T., Lin Y.-M., Valdes-Garcia A., Avouris P. (2009). Ultrafast graphene photodetector. *Nature Nanotechnology*.

[37] Yin Z., Li H., Li H. (2012). Single-layer MoS_2_ phototransistors. *ACS Nano*.

[38] Yuan H., Liu X., Afshinmanesh F. (2015). Polarization-sensitive broadband photodetector using a black phosphorus vertical p-n junction. *Nature Nanotechnology*.

[39] Zheng Z. Q., Zhang T. M., Yao J. D., Zhang Y., Xu J. R., Yang G. W. (2016). Flexible,transparent and ultra-broadband photodetector based on large-area WSe_2_ film for wearable devices. *Nanotechnology*.

[40] Jacobs-Gedrim R. B., Shanmugam M., Jain N. (2014). Extraordinary photoresponse in two-dimensional In_2_Se_3_ nanosheets. *ACS Nano*.

[41] Lembke D., Bertolazzi S., Kis A. (2015). Single-layer MoS_2_ electronics. *Accounts of Chemical Research*.

[42] Ji Q., Zhang Y., Gao T. (2013). Epitaxial monolayer MoS_2_ on mica with novel photoluminescence. *Nano Letters*.

[43] Splendiani A., Sun L., Zhang Y. (2010). Emerging photoluminescence in monolayer MoS_2_. *Nano Letters*.

[44] Guo L., Yan H., Moore Q. (2015). Elastic properties of van der Waals epitaxy grown bismuth telluride 2D nanosheets. *Nanoscale*.

