# Supplementary material for: Confined van der Waals Epitaxial Growth of Two-Dimensional Large Single-Crystal In2Se3 for Flexible Broadband Photodetectors
Source: Research (Wash D C). 2019 Mar 19;2019:2763704. doi: 10.34133/2019/2763704 (PMC6750059; doi:10.34133/2019/2763704)
Supplement: Supplementary Materials — Figure S1. Crystal structure of layered In2Se3 with each layer composed of Se-In-Se-In-Se atomic sheets. Figure S2. Schematics showing viscous laminar flow in the vapor deposition process and related physical parameters. Figure S3. Thermo-gravimetric analysis of the In2Se3 source in an Ar atmosphere, and the temperature window for the growth of 2D In2Se3 in this work is shown by the green region. Figure S4. AFM images of the as-grown 2D In2Se3 on mica by confined growth. Figure S5. Survey XPS spectrum of as-grown 2D In2Se3 on mica. Figure S6. XRD patterns of 2D In2Se3 grown on mica (red) with reference patterns from a blank mica substrate (blue), bulk In2Se3 (green), and a simulated diffractogram (black). Figure S7. PDMS assisted transfer of 2D In2Se3 from a mica substrate onto different substrates. Figure S8. Time-resolved photoresponse of the 2D In2Se3 photodetector under 850 nm and 940 nm light. Figure S9. I–V curves of the 2D In2Se3 photodetector under 660 nm incident light with different power values. Figure S10. I–V curves of the 2D In2Se3 photodetector under different incident light wavelengths. [file 2763704.f1.docx]

Supplementary Materials for *Research*

# Confined van der Waals Epitaxial Growth of Two-Dimensional Large Single-Crystal In_2_Se_3_ for Flexible Broadband Photodetectors

Lei Tang,^†&^ Changjiu Teng,^†&^ Yuting Luo,^†^ Usman Khan,^†^ Haiyang Pan,^‡^ Zhengyang Cai,^†^ Yue Zhao,^‡ §^ Bilu Liu^*†^ and Hui-Ming Cheng^*†#^

^†^Shenzhen Geim Graphene Center, Tsinghua-Berkeley Shenzhen Institute, Tsinghua University, Shenzhen 518055, P. R. China.

^‡^Shenzhen Institute for Quantum Science and Engineering and Department of Physics, Southern University of Science and Technology, Shenzhen 518055, P. R. China.

^§^Shenzhen Key Laboratory of Quantum Science and Engineering, Shenzhen 518055, P. R. China,

^#^Shenyang National Laboratory for Materials Sciences, Institute of Metal Research, Chinese Academy of Sciences, Shenyang 110016, P. R. China.

^&^These authors contributed equally.

Correspondence should be addressed to [bilu.liu@sz.tsinghua.edu.cn](mailto:bilu.liu@sz.tsinghua.edu.cn,)

[hmcheng@sz.tsinghua.edu.cn](mailto:hmcheng@sz.tsinghua.edu.cn)

**Table S1**. Comparisons of the domain sizes of 2D In_2_Se_3_ with other 2D materials.

| **2D materials** | **Domain sizes** | **Methods/Strategies** | **Ref.** |
| --- | --- | --- | --- |
| Graphene | meter | Epitaxial CVD growth on industrial Cu foil | ^[1]^ |
|  | centimeter | CVD growth on melamine pretreated copper surface | ^[2]^ |
|  | millimeter | Ambient-pressure CVD growth on Pt foil | ^[3]^ |
| TMDC | 350 μm | Oxygen-assisted CVD growth on c-face sapphire | [4] |
|  | millimeter | Ambient-pressure CVD growth on Au foil | [5] |
|  | 835 μm | PVD growth on SiO_2_/Si | [6] |
| h-BN | 330 μm | Water-assisted CVD growth on liquid Cu surface | [7] |
|  | 300 μm | CVD growth on enclosure Cu foil | [8] |
|  | 130 μm | CVD growth on binary Cu–Ni alloy | [9] |
| In_2_Se_3_ | 10 μm | Atmospheric pressure PVD growth on SiO_2_/Si | [10] |
|  | 10 μm | van der Waals epitaxy growth on mica | [11] |
|  | 40 μm  40 μm  >200 μm | van der Waals epitaxy growth on mica  Growth in conventional reactor  Growth in confined micro-reactor | [12]  **This work**  **This work** |

**Figure S1**. Crystal structure of layered In_2_Se_3_ with each layer composed of Se-In-Se-In-Se atomic sheets. The thickness of monolayer In_2_Se_3_ is about 1.0 nm.

**Figure S2**. Schematics showing viscous laminar flow in the vapor deposition process and related physical parameters. (a) Schematic showing the formation of a stagnant layer above the substrate surface. Here, *σ* is the average thickness of the stagnant layer, L is the length of the substrate, and V_0_ is the flow velocity. (b) Schematic showing the absorption, migration, and desorption of the flux, where *J*_1_ is the flux to the surface and *J*_2_ is the reaction flux.

As shown in Figure S2, in the vapor phase deposition process the gas flow passing the near surface of the substrate has a gradient distribution of velocity due to the formation of a stagnant layer^[13, 14]^

*σ* = $\frac{10}{3} \frac{L}{\sqrt{\mathrm{Re}}}$ (S1)

where *σ* is the average width of the stagnant layer, and L is the length of the substrate. Re is the Reynolds number of the flow, which is a measure of the type of flow and is given by the follow equation,

Re = *ρvd*/*γ* (S2)

where *ρ* is the flow density, *v* is the flow velocity, *γ* is the coefficient of viscosity of the flow, and *d* is the characteristic linear dimension of the reactor. If Re is greater than 2000, the flow is turbulent, but if it is smaller than 10, the flow is laminar.

In addition, the gas-phase mass transport coefficient (*h*_g_) is given by,

*h*_g_ = $\frac{3}{2}\frac{D}{L}\sqrt{\mathrm{Re}}$ $\frac{D}{<>}= \frac{3}{2}\frac{D}{L}\sqrt{\mathrm{Re}}$ (S3)

where *D* is the mass diffusion constant .

As a result the flux to the surface (*J*_1_) and the reaction flux (*J*_2_) can be expressed as follows,

*J*_1_ = *h*_g_ (*C*_g_-*C*_s_) (S4)

*J*_2_ = *k*_s_*C*_s_ (S5)

where *C*_g_ and *C*_s_ are the concentrations of precursor in the gas phase and on the substrate surface, and *k*_s_ is surface reaction rate.

If the flow is steady state, we have the following result;

*C*_s_ = $\frac{1}{1+\frac{k_{s}}{hg}}$*C*_g_ (S6)

Based on the above analysis, we can deduce a relationship between *C*_s_ and *d*, given by,

*C*_s_ ~ $\frac{1}{1+\frac{k_{s}}{\sqrt{d}}}$*C*_g_ (S7)

In our confined micro-reactor which has a much smaller d, and therefore a much smaller *C*_s_ than in a conventional quartz tube reactor. Therefore, the confined micro-reactor design results in a smaller concentration of precursor on the surface, and consequently fewer nucleation sites. This feature is the foundation for the growth of 2D In_2_Se_3_ with large domain sizes in the confined micro-reactor.

**Table S2**. A list of physical quantity and their definitions.

| **Physical quantity** | **Definition** |
| --- | --- |
| *σ* | the average width of the stagnant layer |
| Re | Reynolds number |
| *L* | the length of substrate |
| *h*_g_ | the gas phase mass transport coefficient |
| *ρ* | the density of flow |
| *v* | the velocity of flow |
| *γ* | the coefficient of viscosity of flow |
| *d* | the characteristic linear dimension of the reactor |
| *D* | the diffusion constant of mass |
| *J*_1_ | the flux to surface |
| *J*_2_ | the reaction flux |
| *C*_g_ | the concentrations of precursor in gas phase |
| *C*_s_ | the concentrations of precursors on substrate surface |
| *k*_s_ | the surface reaction rate |


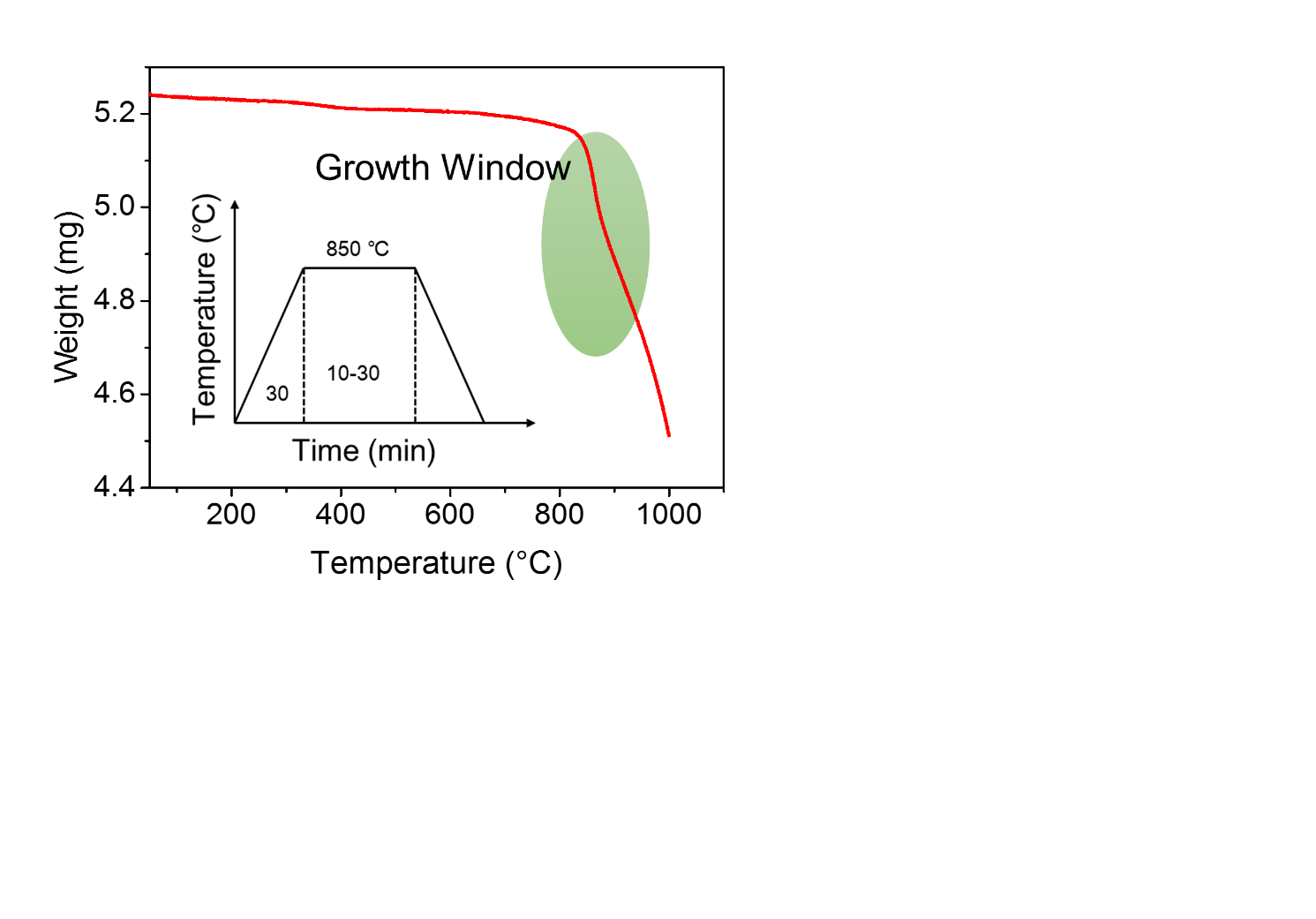


**Figure S3**. Thermo-gravimetric analysis of the In_2_Se_3_ source in an Ar atmosphere, and the temperature window for the growth of 2D In_2_Se_3_ in this work is shown by the green region. The inset is for a typical experiment to grow 2D In_2_Se_3_ at 850 °C.

**Figure S4**. AFM images of the as-grown 2D In_2_Se_3_ on mica by confined growth. The results show that the as-grown 2D In_2_Se_3_ has large domain sizes, a regular triangular shape, and sharp edges.


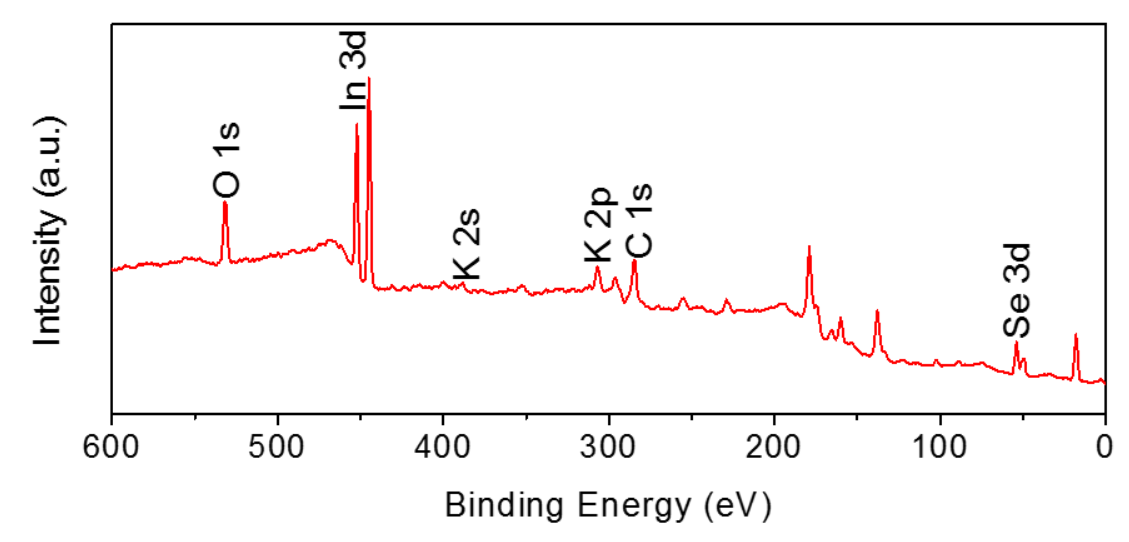


**Figure S5**. Survey XPS spectrum of as-grown 2D In_2_Se_3_ on mica. The result shows an atomic ratio of ~2:3 (9.48%:14.45%) for In and Se elements, suggesting good stereochemistry of the grown 2D In_2_Se_3_. Here, K, O, and C elements are due to the mica substrate or the environment.

**Figure S6**. XRD patterns of 2D In_2_Se_3_ grown on mica (red) with reference patterns from a blank mica substrate (blue), bulk In_2_Se_3_ (green), and a simulated diffractogram (black). The three peaks at 18.47°, 40.68°, and 43.23° can be respectively indexed to the (006), (1,0,10), and (0,1,11) of hexagonal In_2_Se_3_.

**Figure S7**. PDMS assisted transfer of 2D In_2_Se_3_ from a mica substrate onto different substrates. (a) PDMS substrate, (b) SiO_2_/Si substrate, (c) copper TEM grid, and (d) ITO. (e-h) Corresponding typical Raman spectra of the transferred samples under a 532 nm excitation laser.

**Figure S8**. Time-resolved photoresponse of the 2D In_2_Se_3_ photodetector under (a) 850 nm and (b) 940 nm light. The photodetector was bent 1000 times to a radius of 5 mm before the measurements. The results indicate good stability of the 2D In_2_Se_3_ flexible photodetector. The *V*_ds_ is 1 V.

**Figure S9**. I–V curves of the 2D In_2_Se_3_ photodetector under 660 nm incident light with different power values. The results show that current increases with increasing incident power. The *V*_ds_ is 1 V.

**Figure S10**. I–V curves of the 2D In_2_Se_3_ photodetector under different incident light wavelengths. The results show that the photodetector has a broadband response in UV-Vis-NIR range.

**References**

[1] Xu, X.; Zhang, Z.; Dong, J.; Yi, D.; Niu, J.; Wu, M.; Lin, L.; Yin, R.; Li, M.; Zhou, J.; Wang, S.; Sun, J.; Duan, X.; Gao, P.; Jiang, Y.; Wu, X.; Peng, H.; Ruoff, R. S.; Liu, Z.; Yu, D.; Wang, E.; Ding, F.; Liu, K., Ultrafast Epitaxial Growth of Metre-sized Single-crystal Graphene on Industrial Cu Foil. *Scie Bull* **2017,** *62* (15), 1074-1080.

[2] Wu, T.; Zhang, X.; Yuan, Q.; Xue, J.; Lu, G.; Liu, Z.; Wang, H.; Wang, H.; Ding, F.; Yu, Q.; Xie, X.; Jiang, M., Fast Growth of Inch-sized Single-crystalline Graphene from a Controlled Single Nucleus on Cu-Ni Alloys. *Nat Mater* **2016,** *15* (1), 43-47.

[3] Gao, L.; Ren, W.; Xu, H.; Jin, L.; Wang, Z.; Ma, T.; Ma, L. P.; Zhang, Z.; Fu, Q.; Peng, L. M.; Bao, X.; Cheng, H. M., Repeated Growth and Bubbling Transfer of Graphene with Millimetre-size Single-Crystal Grains Using Platinum. *Nat Commun* **2012,** *3*, 699-705.

[4] Chen, W.; Zhao, J.; Zhang, J.; Gu, L.; Yang, Z.; Li, X.; Yu, H.; Zhu, X.; Yang, R.; Shi, D.; Lin, X.; Guo, J.; Bai, X.; Zhang, G., Oxygen-Assisted Chemical Vapor Deposition Growth of Large Single-Crystal and High-Quality Monolayer MoS_2_. *J Am Chem Soc* **2015,** *137* (50), 15632-15635.

[5] Gao, Y.; Hong, Y. L.; Yin, L. C.; Wu, Z.; Yang, Z.; Chen, M. L.; Liu, Z.; Ma, T.; Sun, D. M.; Ni, Z.; Ma, X. L.; Cheng, H. M.; Ren, W., Ultrafast Growth of High-Quality Monolayer WSe_2_ on Au. *Adv Mater* **2017,** *29* (29), 1700990-1700997.

[6] Yang, T.; Zheng, B.; Wang, Z.; Xu, T.; Pan, C.; Zou, J.; Zhang, X.; Qi, Z.; Liu, H.; Feng, Y.; Hu, W.; Miao, F.; Sun, L.; Duan, X.; Pan, A., Van der Waals Epitaxial Growth and Optoelectronics of Large-scale WSe_2_/SnS_2_ Vertical Bilayer p-n Junctions. *Nat Commun* **2017,** *8* (1), 1906-1914.

[7] Wang, L.; Wu, B.; Liu, H.; Huang, L.; Li, Y.; Guo, W.; Chen, X.; Peng, P.; Fu, L.; Yang, Y.; Hu, P.; Liu, Y., Water-assisted Growth of Large-sized Single Crystal Hexagonal Boron Nitride Grains. *Mater Chem Front* **2017,** *1* (9), 1836-1840.

[8] Ji, Y.; Calderon, B.; Han, Y.; Cueva, P.; Jungwirth, N. R.; Alsalman, H. A.; Hwang, J.; Fuchs, G. D.; Muller, D. A.; Spencer, M. G., Chemical Vapor Deposition Growth of Large Single-Crystal Mono-, Bi-, Tri-Layer Hexagonal Boron Nitride and Their Interlayer Stacking. *ACS Nano* **2017,** *11* (12), 12057-12066.

[9] Lu, G.; Wu, T.; Yuan, Q.; Wang, H.; Wang, H.; Ding, F.; Xie, X.; Jiang, M., Synthesis of Large Single-crystal Hexagonal Boron Nitride Grains on Cu-Ni Alloy. *Nat Commun* **2015,** *6*, 6160-6166.

[10] Zhou, J.; Zeng, Q.; Lv, D.; Sun, L.; Niu, L.; Fu, W.; Liu, F.; Shen, Z.; Jin, C.; Liu, Z., Controlled Synthesis of High-Quality Monolayered alpha-In_2_Se_3_ via Physical Vapor Deposition. *Nano Lett* **2015,** *15* (10), 6400- 6405.

[11] Zheng, W.; Xie, T.; Zhou, Y.; Chen, Y. L.; Jiang, W.; Zhao, S.; Wu, J.; Jing, Y.; Wu, Y.; Chen, G.; Guo, Y.; Yin, J.; Huang, S.; Xu, H. Q.; Liu, Z.; Peng, H., Patterning Two-dimensional Chalcogenide Crystals of Bi_2_Se_3_ and In_2_Se_3_ and Efficient Photodetectors. *Nat Commun* **2015,** *6*, 6972-6979.

[12] Lin, M.; Wu, D.; Zhou, Y.; Huang, W.; Jiang, W.; Zheng, W.; Zhao, S.; Jin, C.; Guo, Y.; Peng, H.; Liu, Z., Controlled Growth of Atomically Thin In_2_Se_3_ Flakes by van der Waals Epitaxy. *J Am Chem Soc* **2013,** *135* (36), 13274- 13277.

[13] Bhaviripudi, S.; Jia, X.; Dresselhaus, M. S.; Kong, J., Role of Kinetic Factors in Chemical Vapor Deposition Synthesis of Uniform Large Area Graphene Using Copper Catalyst. *Nano Lett* **2010,** *10* (10), 4128-4133.

[14] Hugh O. Pierson, Handbook of Chemical Vapor Depsition (CVD). Principles, Technology, and Applications. The United States of America by Noyes Publications, **1999**.
